# Overcoming barriers in evaluating outbreaks of diarrheal disease in resource poor settings: assessment of recurrent outbreaks in Chobe District, Botswana

**DOI:** 10.1186/1471-2458-13-775

**Published:** 2013-08-26

**Authors:** KA Alexander, JK Blackburn

**Affiliations:** 1 Department of Fish and Wildlife Conservation, Virginia Tech, 132 Cheatham Hall, Blacksburg, Virginia 24061, USA; 2 CARACAL (Center for Conservation of African Resources: Animals, Communities and Land use), Kasane, Botswana; 3 Department of Geography, Spatial Epidemiology & Ecology Research Laboratory, University of Florida, Gainesville, Florida, USA; 4 Emerging Pathogens Institute, University of Florida, Gainesville, Florida, USA

**Keywords:** Diarrhea, Outbreak investigation, Breastfeeding, Water shortage, Water quality, Surface water, Climate, Under 5 years of age, Cluster analysis, Public health, Poverty, Resource poor, Africa, Botswana

## Abstract

**Background:**

Diarrheal illness remains a leading cause of global morbidity and mortality, with the majority of deaths occurring in children <5 years of age. Lack of resources often prohibits the evaluation of outbreak characteristics and limits progress in managing this important disease syndrome, particularly in Africa. Relying only on existing medical staff and hospital resources, we assess the use of a questionnaire survey tool to identify baseline outbreak characteristics during recurrent diarrheal outbreaks in Chobe, Botswana.

**Methods:**

Using historical surveillance data (2006–2009), the temporal pattern of recurrent diarrheal outbreaks was evaluated among patients <5 years of age presenting to health facilities in Chobe District. Using a questionnaire survey tool, medical staff from selected health facilities assessed patients (all ages) presenting with diarrheal disease during two diarrheal outbreaks (2011–2012). Cluster analysis and classification and regression trees (CART) were used to evaluate patient attributes by outbreak.

**Results:**

We identified a bimodal, annual pattern of acute diarrhea in children <5 years of age across years (Wilcox test, W = 456.5, p = 0.052). Historical outbreak periods appeared to coincide with major hydrological phenomena (rainfall/flood recession). Across health facilities, a significant percent of patients in the prospective study were in the ≥5 age class (44%, n = 515 and 35%, n = 333 in the dry and wet season outbreaks, respectively). Cluster analysis of questionnaire data identified two main branches associated with patient age (<5 and ≥5 years of age). Patients did not cluster by outbreak or village. CART examination identified sex and hospitalization as being most predictive of patients <5 years and household diarrhea in patients ≥5 years. Water shortages and water quality deficiencies were identified in both outbreaks.

**Conclusions:**

Diarrhea is a persistent, seasonally occurring disease in Chobe District, Botswana. Lack of variation in outbreak variables suggests the possibility of environmental drivers influencing outbreak dynamics and the potential importance of human-environmental linkages in this region. Public health strategy should be directed at securing improved water service and correcting water quality deficiencies. Public health education should include increased emphasis on sanitation practices when providing care to household members with diarrhea. While global diarrheal disease surveillance is directed at the under-5 age group, this may not be appropriate in areas of high HIV prevalence such as that found in our study area where a large immune-compromised population may warrant increased surveillance across age groups. The approach used in this study provided the first detailed characterization of diarrheal disease outbreaks in the area, an important starting point for immediate intervention and development of working hypotheses for future disease investigations. While data derived from this approach are necessarily limited, they identify critical information on outbreak characteristics in resource poor settings where data gaps continue and disease incidence is high.

## Background

Diarrheal illness remains one of the leading causes of global morbidity and mortality, with the majority of deaths occurring in children under 5 years of age [1]. The World Health Organization (WHO) estimates that nearly nine million children in this age group die each year, identifying diarrheal disease as one of the most important sources of childhood mortality outside of pneumonia [2]. Despite advances in health care globally, the estimated median incidence of diarrheal disease has not changed significantly for under-5s from the early 1990s to 2003 [3].

The manner in which climate, environment, socioeconomics, behavior, and concurrent disease (e.g., HIV), interact and influence diarrheal disease occurrence is uncertain and difficult to deconstruct [4-13]. Identifying the interdependent manner in which these drivers influence pathogen transmission pathways presents one of the greatest challenges to management of this persistent public health problem.

### Resource poor settings and health research

Nearly half of all child diarrheal deaths occur in Africa (46%, [2]), highlighting the importance of understanding this disease syndrome and its control on the continent. In many places in Africa, remoteness of health facilities and increasing patient burdens limit outbreak investigation capacity and there is a paucity of information (published or not) regarding patient characteristics and risk factors associated with diarrheal disease outbreaks, despite their frequency of occurrence. The human and economic resources required to undertake a full epidemiological study during disease outbreaks are normally not available. Health system reform and strengthening of service provision, including laboratory diagnostic capacity, is a key regional focus [14,15]. Programs have also been developed to increase health surveillance data quality, as for example through the Integrated Disease Surveillance and Response (IDSR) strategy under the World Health Organization African Regional Office (WHO AFRO) in partnership with the United States Centers for Disease Control and Prevention (CDC). However, data derived from passive surveillance systems often suffer from a number of deficiencies and in most cases, only identify case incidence by clinical diagnosis, age category, gender, and treatment outcome (death or discharge, for a full review of passive surveillance limitations see [7]). Other sources of information are generally unavailable in these resource poor settings, characterizing much of Africa, particularly those in more remote regions distant from primary medical facilities.

Hospitals and clinics in much of Africa are understaffed with limited resource support where disease outbreaks tax already inadequately staffed medical facilities. For example, in Chobe District, Botswana (a now middle income country), there were only 9 government doctors (Table 1) available to serve the entire district population of 23,347 [16]. In such situations, the primary public health focus is directed at treating the sick and managing hospital functions (including disease surveillance reporting) and often there are no resources available to prospectively investigate outbreak dynamics. Certainly, there is limited ability to identify control groups and characterize risk factors during an outbreak. Thus, little progress is made in understanding this disease syndrome and identifying appropriate public health interventions. Indeed, when evaluating the number of diarrheal outbreak investigations published in peer reviewed journals from 1948 (some journals date back further) to 2012 in the PubMed database [17] using the search terms, “diarrhea,” “outbreak,” and the location of interest (e.g., Botswana, Namibia), there is an extremely limited amount of published information (Table 2). In Namibia, for example, we could not find any publications with these search terms. In other countries, such as Botswana, the few studies that were identified in the database were conducted with assistance from public health officials from the CDC. The absence of published data is stark when compared to the number of publications in a developed country, such as the United States, using the same terms (n = 359) where greater financial and human resource support is available for outbreak investigations. How do we overcome this barrier?

**Table 1:** **Health personnel staffing Botswana Government health facilities in Chobe District by sex and category**[25]

Health personnel by category^*^	**Sex**	**Total**
Medical doctors	8M, 1F	9
Dentist	1M	1
Dental therapist	1M	1
Dental assistant	1M	1
Senior nursing sisters	19M, 35F	54
Registered nurse	13M, 25F	48
Hospital orderly	1M, 15F	16
Health care auxiliary	2M, 22F	24
Social workers technicians	2M, 2F	4
Medical technologist/scientist	1M	1
Medical laboratory technicians	5M	5
Medical laboratory assistant	1M	1
Radiographer technician	2M	2
X-Ray attendant	1M	1
Pharmacy technicians	7M, 1F	7
Dietician and health education officers	1M, 1F	2
Medical records filing clerk	1M, 1F	2

*As reported in 2009, non-medical support staff are not included.

**Table 2:** **Number of journal articles identified in the PubMed database**[17]**using the search terms “outbreak”, “diarrhea”, and the country of interest**

**Location**	**Search terms:**	**Referenced publications**
	outbreak and diarrhea*	
Botswana	3	[32,41,47]
Namibia	0	0
Zambia	4	[64-67]
Zimbabwe	9	[68-76]
South Africa	15	[77-91]

^*^Papers retrieved with these key words were included in the publication count from PubMed database whether or not they reflected an investigation of a primary diarrheal outbreak*.*

In Botswana, as elsewhere in Africa, diarrheal disease is a leading cause of morbidity and mortality, particularly in children [18]. Botswana also has one of the highest HIV prevalence levels in the world [19] with an estimated national prevalence of 17.6% in 2008 [20]. This epidemic has impacted population vulnerability to infectious disease generally and diarrheal disease in particular [21]. High diarrheal incidence in the country has been attributed to a variety of factors such as consumption of contaminated water (including preparation of baby foods), unhealthy environmental conditions such as poor hygiene, and inadequate human waste disposal facilities [22], although empirical data are limited. Chobe District, in Northern Botswana, is located in a remote area of the country far removed from primary urban centers, central human resources, and infrastructure. Due to institutional limitations, data are only generally collected on the summary diagnoses, demographics, and treatment outcomes. This is, however, inconsistently achieved. Diagnoses are predominately clinical in nature due to restricted capacity for diagnostic assessments in relation to limited staffing and expansive diagnostic workloads related in part to the HIV/AIDS epidemic [14].

In this study, we evaluated the temporal patterns of recurrent annual diarrheal outbreaks in under-5s in Chobe District, Botswana from 2006–2009. Using a questionnaire based survey tool, we prospectively assessed patient characteristics during two diarrheal outbreaks occurring in 2011–2012 and identified primary attributes of affected individuals. We developed this questionnaire-based approach specifically to be used by existing medical staff within existing institutional limitations to provide insight into the characteristics of outbreaks. We assess the application of this approach and applied exploratory tools (cluster analysis, recursive partitioning) in initial outbreak assessments in resource poor areas and evaluate their role in directing future research and management of diarrheal disease outbreaks.

## Methods

### Study site

**Figure 1:**
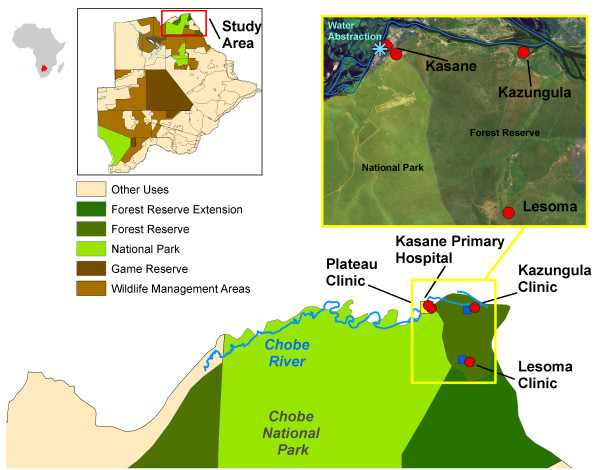
**Study area map.** Map of villages reporting cases during diarrheal outbreaks (red dots) with the Kasane hospital (yellow square) and clinics (blue squares). Chobe District is a multi-land use area comprised of both protected (green areas) and unprotected areas (tan areas). The Chobe River (blue line) traverses both of these land types before abstraction for municipal use (iblue star).

Botswana is a politically stable, semi-arid, landlocked country located in sub-Saharan Africa. The study area is located in Chobe District, the northernmost district in the country (Figure 1). The District population is spread across two urban communities and seven smaller villages. The country has a subtropical climate with annual wet (November-March) and dry (April-October) seasons. Rainfall is extremely variable in the country both within and between years with recurrent occurrence of both flooding and droughts [23]. The Chobe River, the primary source of all municipal water in the District (with exception of one village) floods annually [24]. Piped water is available either through direct reticulation to residences or limited provision of water through public taps.

Government health services (health posts, clinics, and hospitals) are available to the public for a nominal charge (<1 US dollar). The District has one primary hospital, three clinics, and 12 health posts serving the entire population. Distance to medical facilities is lower than the rural average in Botswana with 60% living within 5 km, 31% living within 5–8 km, and 9% living within an 8–15 km radius of a health facility [25]. Kasane Primary Hospital was built in 1962, four years before independence from British rule and with some limited upgrading and temporary building additions, the main facility is relatively unchanged. The hospital has a limited capacity of 29 beds across all wards (maternal (6), pediatrics (7), general wards (12), and tuberculosis isolation unit (4))[25]. Medical staffing is presented in Table 1 and is extremely limited relative to the population of the District.

### Retrospective assessment of seasonal dynamics of diarrheal disease in children under five (2006–2009)

The Botswana Integrated Disease Surveillance and Response (IDSR) Program monitors the case incidence of diarrhea among children under or equal to 5 years of age (under-5) presenting to clinics and hospitals across administrative Districts of Botswana. Diarrheal disease is defined as the occurrence of at least 3 loose stools in a 24-hour period within the four days preceding the health visit. In order to account for reporting bias associated with bioclimatic or socioeconomic factors over time, we evaluated the data as the proportion of monthly deviation from the mean of monthly diarrheal case incidence for the respective year. Our data were, thus, standardized on a yearly basis, transforming the data as follows:

$$P_{m,y}=\left(D_{m,y}-\mathrm{Av}e_{y}\right)/\mathrm{Av}e_{y}$$

where *P*_m, y_ is defined as the monthly proportion deviation from the mean number of cases of diarrhea for month (*m*) in year (*y*). *D*_*m, y*_ is the average number of reported cases of diarrhea in month *m* of year *y,* and *Ave*_*y*_ is the mean number of cases of diarrhea by month for year *y*.

### Prospective assessment of patient characteristics (all ages) during two diarrhea outbreaks (2011–2012)

Questionnaires were used to evaluate patients (all ages) presenting with diarrhea to study medical facilities within the District from August 2011-March 2012 and administered by medical staff. Project staff visited the Kasane Government Hospital and participating health clinics (Kazungula, Plateau, and Lesoma) weekly. Survey tools were prepared in both English and Setswana (both National languages) and all human associated data were anonymised. The research was conducted under permit from the Ministry of Health in Botswana and approval from the Virginia Tech Institutional Review Board (IRB# 11–573). Data on diarrheal disease causation were not available as most cases are not diagnostically assessed and treatment regimes were directed at symptomatic care. Questionnaires were used to characterize only those patients presenting with diarrheal disease and was not used outside the hospital/clinic setting on unaffected individuals.

### Fecal microorganism data Chobe District

Stool samples were submitted to the Kasane Primary Hospital laboratory for diagnostic purposes as well as health certification for employment. Data (August 2007- October 2011) on organism identification were manually extracted from laboratory books.

### Statistical analysis

To assess the seasonal occurrence of diarrhea in children under-5, we used a one-way analysis of variance (ANOVA). Months were categorized as diarrheal outbreak months (January - March, July - October) or non-outbreak months (April-June, November, December) in the dry and wet season respectively, based on the knowledge of hospital staff and local understanding of months associated with annually recurrent diarrheal outbreaks during these seasons. Differences in the two samples were compared using the Mann–Whitney–Wilcoxon (MWW) test [26]. Independence of data points was assumed.

Data from each diarrheal outbreak were extracted from questionnaires. Descriptive statistics and frequency tables were computed for each outbreak and age category using SPSS version 20 (IBM Inc., Armonk, NY). Exact binomial 95% confidence intervals were calculated using the Epitools package in R [27].

Data from select questions were then combined for cluster analysis and recursive partitioning to explore differences in patient characteristics between outbreaks and dominant divergent characteristics of patients presenting with diarrheal disease. Twelve variables were used in the analysis with responses being nominal (village: Kasane, Kazungala, Plateau, or other; breast fed: yes, no or not applicable) and binary, symmetrical variables (sex, age, hospitalized, boiling of water, presence of dirt in council water, flush toilet, occurrence of household diarrhea, having water piped indoors, water storage, and the occurrence of water shortages). These variables were selected from the larger group based on their ability to provide insight into the demographics of the assessed population and characteristics of the outbreak. The use of village allowed us to assess whether there was an explicit spatial component to the outbreaks. The add-on package Cluster and the Daisy algorithm were used in R-statistics [28] for hierarchical cluster analysis, and creation of cluster dendrograms. We used the general dissimilarity coefficient of Gower as our linkage method [29]. It has the advantage of being able to include different types of data in the analysis (nominal, ordinal, and (a)symmetric binary). For comparative purposes, we assessed a number of distance measures (ward, single, average, complete, centroid, and mcquitty) and compared goodness of the dendrogram fit with the dissimilarity matrix using both the Gower Distance Assessment and Cophenitic Correlation [30]. The number of responses varied to limited degree by variable as patients either elected not to answer a question or medical staff did not ask/record the response (missing values in a row of x are not included in the dissimilarities involving that row). Variables that had low response levels such as HIV/AIDS status were not included in the analysis.

Dendrograms were pruned at the level where the major cluster groups appeared to form and could be seen on a clustering heat map where associations are visualized along a color gradient [31]. Dendrograms are scaled by the average distance function representing differences between fusion points in the tree. Recursive partitioning was used to create decision trees that attempt to correctly classify members of clusters by attribute.

## Results

### Diarrheal disease in children under five year olds, 2006–2009

**Figure 2:**
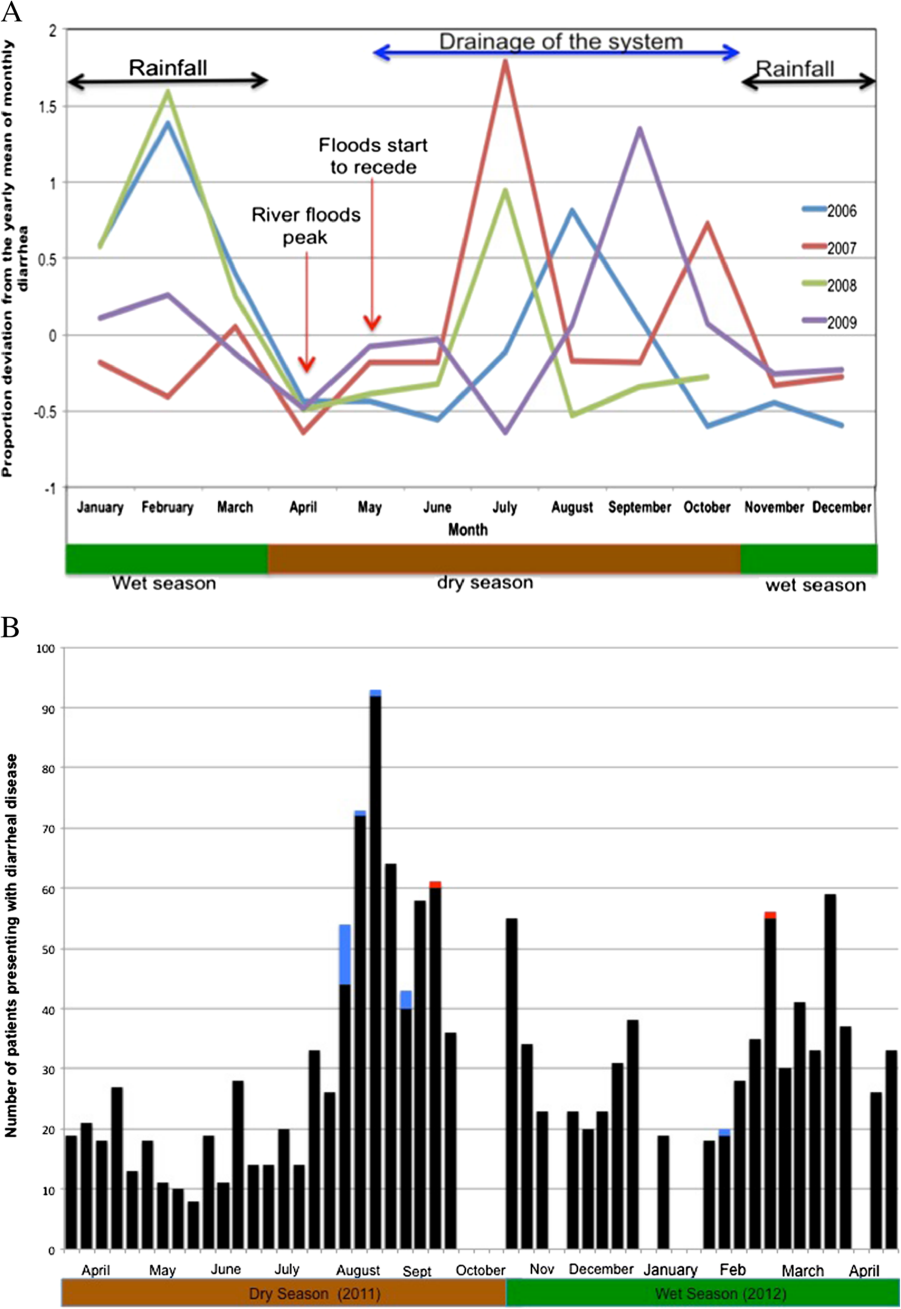
**Diarrheal disease outbreaks in Chobe District, Botswana. A** - Diarrheal disease reported among patients under-<5 years of age presenting to health facilities in Chobe District from 2006–2009. **B** - Number of patients (all ages) presenting to health facilities with diarrheal disease in Chobe District during the study period by week from April 2011 to April 2012 (black - outpatient, blue - hospitalized, red - deaths). Gaps represent weeks where diarrheal incidence data were unavailable.

Historically, diarrheal outbreaks occurred annually from 2006–2009 in the Chobe District with two primary outbreak periods identified by medical staff and local knowledge: January through March during the rainy season and July through October, after the floods began to recede in the water system (Figure 2A). When analyzing diarrhea incidence by month, no differences could be detected (ANOVA, F (11, 33) = 1.65, p = 0.128). However, there was a marginally significant difference in diarrheal incidence between months classified as outbreak months corresponding to perceptions of historical outbreak periods and non-outbreak months in the dry and wet season respectively (Wilcox test, W = 456.5, p = 0.052).

### Fecal organism identification

Organisms identified in stool samples assessed by the Kasane Primary Hospital are presented in Table 3.

**Table 3:** Fecal microorganisms identified in stool samples submitted to the Kasane primary hospital laboratory, Chobe District, Botswana from August 2007 to October of 2011

**Organism**	**Number of times reported**
*Ascaris lumbricoides*	3
*Balantidium coli*	1
*Cryptosporidium* spp	1
*Escherichia coli*	21
*Giardia lamblia*	2
*Klebsiella* spp	2
*Salmonella* spp	17
*Salmonella typhi*	3
*Shigella* spp	3
*Staphylococcus aureus*	1
*Strongyloides stercoralis*	2
*Taenia* spp	5
*Trichuris trichiura*	1

### Questionnaire Survey

Questionnaires were collected from medical staff from August 1-September 30, 2011 (Dry season outbreak) and January 1 through-April 3, 2012 (Wet season outbreak). Of patients presenting with diarrheal disease (all ages), 23% and 19% were interviewed by medical staff in the study medical facilities in the dry and wet season outbreaks respectively (Table 4).

**Table 4:** Percentage of patients surveyed by age group and diarrheal outbreak season at the selected study health facilities (Kasane Primary Hospital, and Plateau, Kazungula and Lesoma Clinics) in Chobe District, Botswana 2011–2012

	**Patients presenting with diarrheal disease at study facilities**			**Percentage surveyed**		
Outbreak period	Patients <5	Patients ≥5	Total patients	Patients <5	Patients ≥5	Total patients
Dry season (2011)	153	69	222	18%	32%	23%
Wet season (2012)	75	62	137	27%	10%	19%

### Outbreak characteristics 2011–2012

**Figure 3:**
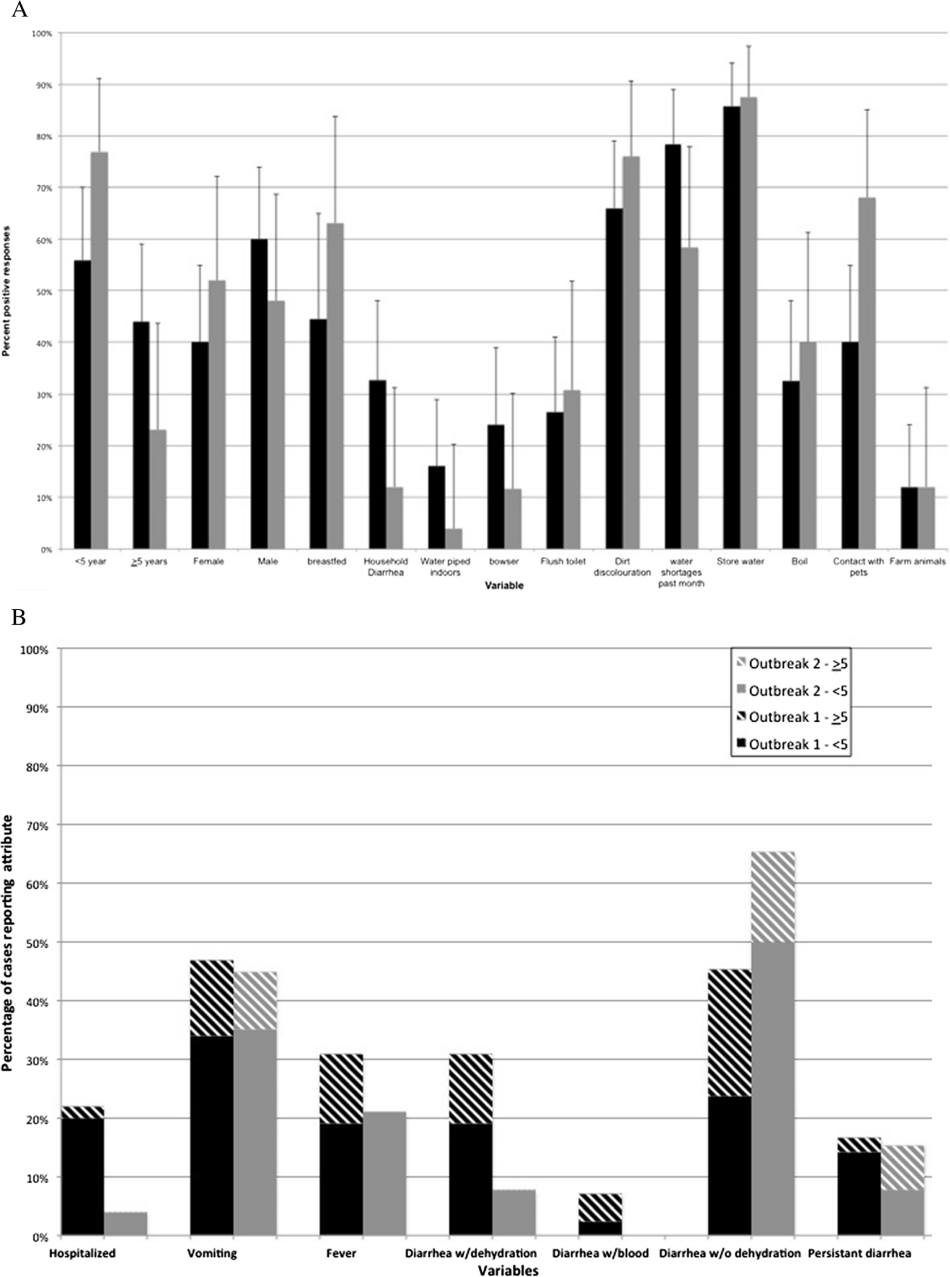
**Comparison of clinical and patient characteristics during two diarrheal outbreaks in Chobe District, Botswana (2011–2012). A** - Comparison of respondent characteristics in outbreak 1 (dry season 2011, black) and outbreak 2 (wet season 2012, grey) in Chobe Botswana. **B** - Comparison of clinical features of diarrheal patients from questionnaire data by age and outbreak in Chobe District, Botswana (2011–2012). Confidence intervals on each variable are identified in Table 6.

Patient demographics and diarrheal disease type are identified in Table 5 and include all diarrhea patients presenting to medical facilities and mobile health stops in the District by outbreak period. The number of under-5′s presenting with diarrheal disease from April 2011 to April 2012 is presented in Figure 2B. Questionnaires were obtained from medical staff at health facilities in August (n = 47), September (n = 3), January (n = 3), February (n = 14), and March (n = 9) during this time period. Attributes identified from questionnaires by outbreak are presented in Figure 3A and clinical presentation by age and outbreak are illustrated in Figure 3B (95% CIs presented in Table 6). No significant differences could be identified between variables when compared by outbreak period. Questionnaire derived data were not analyzed by village because of insufficient sample size.

**Table 5:** Numbers of patients by age, sex, and diarrhea type presenting at health facilities and mobile stops in Chobe District, Botswana during study out break periods in 2011–2012

**Outbreak period**	**Diarrhea without dehydration**						**Diarrhea (acute) with some dehydration**						**Diarrhea (acute) with severe dehydration**						**Diarrhea with blood**						**Total**
	**<1**		**1- 4**		**5 +**		**<1**		**1- 4**		**5 +**		**<1**		**1- 4**		**5 +**		**<1**		**1- 4**		**5 +**		
	**M**	**F**	**M**	**F**	**M**	**F**	**M**	**F**	**M**	**F**	**M**	**F**	**M**	**F**	**M**	**F**	**M**	**F**	**M**	**F**	**M**	**F**	**M**	**F**	
Dry (2011)	34	35	13	18	23	16	39	49	32	30	42	49	29	19	18	13	10	26	30	30	40	40	20	40	515
Wet (2012)	31	36	16	21	34	28	23	20	17	16	16	27	3	3	6	3	1	4	6	5	3	8	4	2	333

**Table 6:** Clinical presentation by age group and outbreak period in Chobe District Botswana (2011–2012)

**Variable**	**Dry season outbreak**	**Wet season outbreak**
Hospitalized <5	36% (19–56%)	5% (0–25%)
Hospitalized ≥5	5% (0–23%)	0% (0–52%)
Vomiting <5	62% (41–80%)	47% (21–73%)
Vomiting ≥5	29% (11–52%)	40% (5–85%)
Fever <5	33% (16–55%)	27% (8–55%)
Fever ≥5	28% (10–53%)	0% (0–60%)
Diarrhea w/dehydration <5	32% (15–54%)	10% (1–32%)
Diarrhea w/dehydration ≥5	29% (10–56%)	0% (0–46%)
Diarrhea w/blood <5	4% (0–20%)	0% (0–17%)
Diarrhea w/blood ≥5	12% (1–36%)	0% (0–46%)
Diarrhea w/o dehydration <5	40% (21–61%)	65% (41–85%)
Diarrhea w/o dehydration ≥5	53% (28–77%)	67% (22–96%)
Persistent diarrhea <5	24% (9–45%)	10% (1–32%)
Persistent diarrhea ≥5	6% (0–29%)	33% (4–78%)

Exact binomial 95% confidence intervals are reported in parentheses.

In outbreak 1, direct exposure to surface water through swimming (6%, 95% CI 1-17%), cleaning vegetables (4%, 95% CI 0-14%), and washing laundry (4%, 95% CI 0-16%) appeared limited. No one reported using the river directly as a primary water source. In outbreak 2, there were no reports of direct river water usage among any of the respondents (n = 26). Among the under-5′s, measles vaccination coverage was reported at 75% and 100% (95% CIs 54-96%, 75-100%, outbreak 1 and 2), respectively. A limited level of malnutrition was reported in outbreak 1 among this same group (14%, 95% CI 3-35%). There were no reports of malnutrition in outbreak 2 (n = 18). HIV positive status was reported at low levels with only 6 positive patients in outbreak 1 (29 negative, and 15 unknown responses, n = 46) and only 2 reporting a positive status in outbreak 2 (11 negative, and 12 unknown responses, n = 25). Attendant nursing staff reported that respondent reluctance to disclose accurate status might have influenced the nature of the response. Reported deaths were low with one under-5 outpatient dying each outbreak period.

### Cluster and classification and regression trees

**Figure 4:**
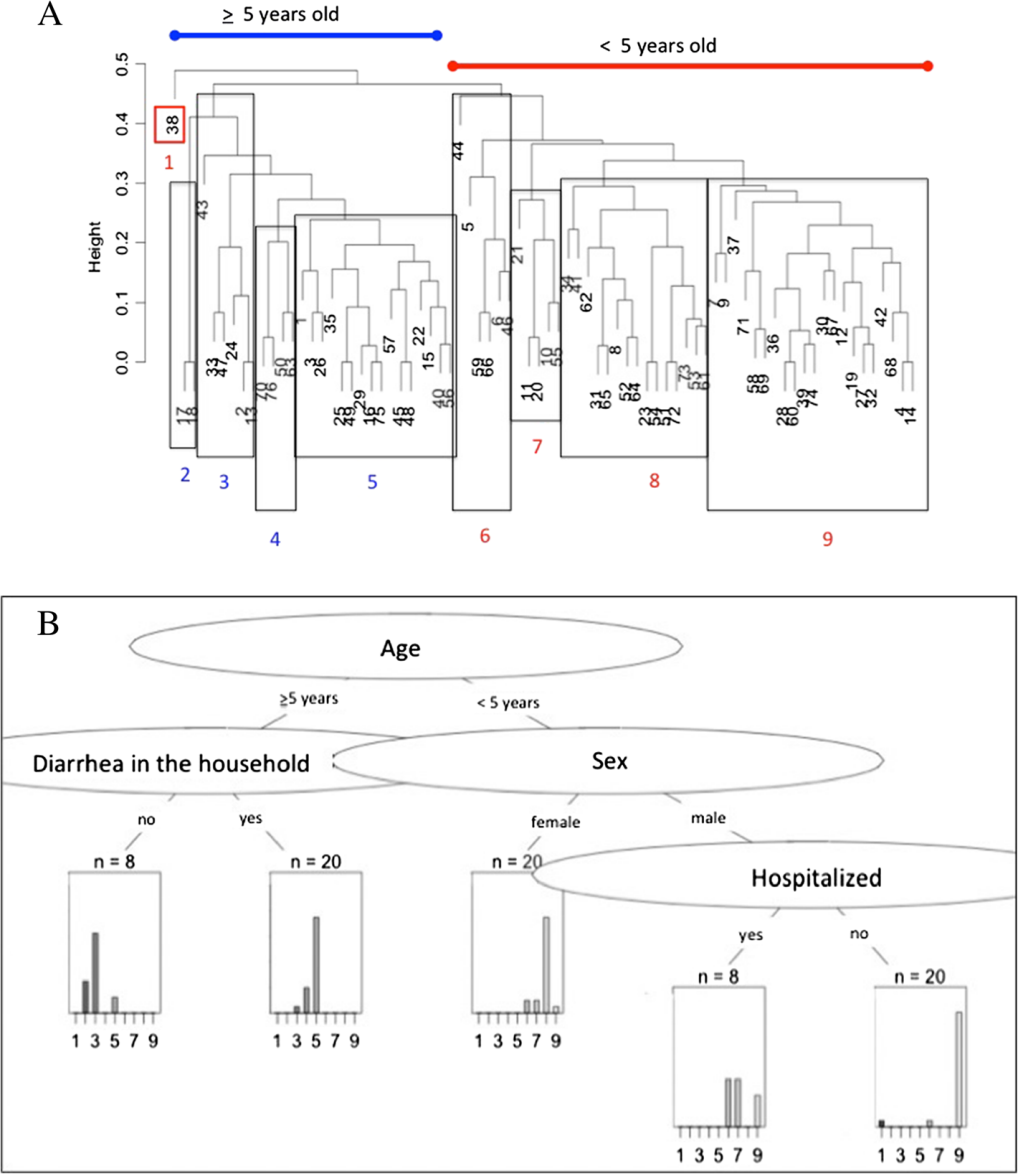
**Cluster and CART analysis of patient characteristics involved in diarrheal outbreaks in Chobe District, Botswana (2011 – 2012). A** - Dendrogram of cluster analysis of questionnaire data collected from patients (blue ≥5 years old, and red <5 years old) presenting to health facilities in Chobe District (2011–2012) during diarrheal outbreaks. The dendrogram was constructed using Gower Dissimilarity linkage method and Average Distance metric. With the exception of patient 38, <5 years olds clustered separately from adults and ≥5 - 14 year olds (n = 3). **B** – Decision tree from the CART analysis of cluster assignments.

The average distance metric provided the best fit to the dissimilarity matrix on both the Gower Distance Assessment and the Cophenetic Correlation. The cluster analysis of questionnaire data identified two main branches representing patients under or equal to five years of age (under-5) and greater than or equal to five years of age (5+). Eight sub-clusters were identified (Figure 4A). Finer level clustering might have been obscured in heat map defined clusters but the focus of the exercise was to identify dominant differences among patients. The CART examination of cluster assignments (Figure 4B) identified sex and hospitalization as being most predictive of patients under-5 and the occurrence of household diarrhea being most important in cluster assignments for patients 5+ (classification error = 0.363). Home village was not a significant discriminatory variable in cluster analyses.

## Discussion

We evaluated the temporal dynamics of reported diarrheal disease and characteristics of patients presenting to select health facilities during outbreak periods in Chobe District, Botswana. The questionnaire survey tool was administered by existing medical staff in attendance and provided important data that would otherwise not have been available in this remote and resource poor setting. To our knowledge, this is the first prospective assessment of diarrheal patient characteristics in this District. Cases of diarrhea were identified among all villages in the District. Death occurred in both outbreaks involving outpatient children under-5 (n = 1 each outbreak). Although medical staffing of health facilities in the District was extremely limited (Table 1), a reasonable proportion of patients were surveyed during outbreak periods despite higher caseloads relative to the outbreak (dry season = 23% and wet season = 19% surveyed, Table 4). We discuss these study results and implications to diarrheal disease control. We then evaluate the effectiveness and limitations of study approaches and their application in resource poor settings.

### Seasonality of diarrheal disease

Our examination of passive surveillance data for the region identifies a seasonal pattern of acute diarrhea in under-5 across years that coincides with major hydrological phenomena occurring seasonally in the area (rainfall and flood recession, Figure 2A). Diarrheal case incidence among this same age group during our study in 2011–2012 was similar in temporal pattern (Figure 2B). This pattern of diarrheal disease at the level of Chobe District appears to diverge from the national pattern [7] where diarrheal case incidence (1974–2003) peaks in March and October. Botswana is an arid country with limited surface water. Villages in the study area with the exception of Pandamatenga rely principally on municipal water obtained from the Chobe River. Potential interactions between rainfall and flooding effects on surface water quality and human diarrheal disease would be an influence limited to those areas where surface water occurs and may be associated with divergence in patterns of diarrheal case incidence. While the temporal pattern of case incidence suggests a strong climatic signature to diarrheal disease in Chobe District, the relative influence of various meteorological variables is unknown. Weather events, however, can strongly influence diarrheal disease as seen before in eastern Botswana in 2006 where unusually heavy rains, flooding, and apparent overflow of pit latrines precipitated a 25-fold increase in mortality with more than 547 under-5 children dying during an associated diarrheal outbreak [32].

### Variation in causality by outbreak

We were interested in understanding whether patient characteristics varied by season of outbreak, as this would provide insight into causality. Responses, however, did not vary significantly by outbreak period (Figure 2A). This is particularly important given the potential for nonrandom patient selection affects, individual variation in the interpretation of questions and patient answers by nursing staff, and limited sample sizes. Furthermore, on cluster analysis, patients did not group by outbreak period (wet or dry season) or village but rather by age, with two distinct clades representing those patients under-5 and those patients 5+ (Figure 3). Lack of variation in patient characteristics by outbreak and seasonal patterns of occurrence support the hypothesis of environmental drivers influencing outbreaks in this region.

Using a decision tree approach, membership in the eight identified clusters was related dominantly to age with patient gender and hospitalization differentiating among patients under-5 and diarrhea in the household the most important varying characteristic among patients 5+ (Figure 4). Variable selection was, by necessity, limited and may not have included key variables important to identifying other important divergent qualities among patients involved in these 2 outbreaks. These results suggest, however, that secondary household transmission may be an important characteristic in adult diarrheal disease and that interventions that dampen these within household transmission pathways may contribute to a reduction in adult diarrheal disease in the study area. For example, improper disposal of children’s feces by the mother or caregiver can contribute importantly to secondary household transmission of diarrhea causing pathogens. A recent national survey found that only 49% of rural households disposed of children’s feces safely [33]. Public health messages and education programs directed at improving hygiene and sanitation practices among mothers and caregivers may strongly contribute to a reduction in secondary household transmission and diarrheal disease.

### Clinical presentation, pathogens, and diarrheal disease

A history of bloody diarrhea was reported at low levels in the first outbreak only, but there were no significant differences in the occurrence of this feature between outbreaks when passive surveillance data were evaluated for this same time period for all ages, health facilities, and mobile health visits in the District (overall 3.9% and 5%, dry and wet season respectively, Table 5). As might be expected, characteristics that occur at very low levels may not be accurately detected given potentially limited sample sizes, which should be considered when applying this approach. This type of clinical information, however, can be very valuable as a number of important bacterial pathogens can cause bloody diarrhea. These include *Salmonella spp.*, *Campylobacter jujuni*, *Shigella spp.,* and enterohemorrhagic and enteropathogenic *Escherichia coli*[34]*,* some of which were identified previously among patients presenting to the primary hospital or clinics in the District (Table 3). Concurrent immunosuppressive conditions (e.g., HIV/AIDS) can increase the severity of disease as well as the occurrence of bloody diarrhea [34]. The low frequency of this clinical sign suggests that these pathogens did not play a dominant role in outbreak occurrence and case presentation.

Enteric viral pathogens can also contribute to diarrheal disease and their contribution to outbreak dynamics is uncertain. Rotavirus is an important seasonal pathogen causing acute diarrheal outbreaks over much of the globe [35]. As with many of the enteric bacterial pathogens, rotavirus can also be transmitted in sewage polluted waters [36]. Pathogen invasion dynamics can be influenced by local climatic drivers including low temperatures and rainfall as well as flooding (reviewed [35]), features of the early dry season in the Chobe area. A survey of enteric viral pathogens among children presenting with diarrhea in Gaborone in southern Botswana identified low levels of infection with rotavirus (9.2%) peaking in July during that study [37]. The impact of this pathogen on diarrheal incidence is expected to be reduced with the Government’s incorporation of the Rotavirus vaccine to the under five immunization schedule in July of 2012 [38]. Measles in Africa also occurs in the dry season [39] and can cause diarrhea. Vaccination coverage for measles in Botswana is estimated to be high (94%) and case identification low (n = 8, 2010), although large outbreaks have occurred [40]. In this study, vaccination coverage was reported at a lower level in the dry season (75% dry season and 100% wet season outbreaks).

Protozoal parasites, such as *Cryptosporidium,* have previously been implicated in diarrheal disease in Botswana in association with a severe outbreak of diarrhea in the eastern part of the country (60%, n = 75 samples tested, [41]). Surprisingly, of the samples collected from patients presenting with diarrhea in the wet and dry season outbreaks evaluated here, only low levels of *Cryptosporidium* were identified (wet season outbreak 25%, n = 12, 95% CI 5% - 57%, dry season outbreaks 3%, n = 30, CI 95% 0% - 17% [42]). Positive cases were seen among children less than two years of age. In Chobe District, as elsewhere, diarrheal causation is complex and likely involves a number of bacterial, parasitic, and viral pathogens, involving a potential diversity of hosts including zoonotic sources and interdependent transmission pathways.

### HIV/AIDS

While Botswana has one of the highest prevalence rates of HIV in the world [19], Chobe District is identified as one of the top five highest affected Districts within the country (13.1% and 30% among men and women respectively, both sexes = 23%, 2008 [20]). HIV/AIDS can have an important influence on immunological competence and host susceptibility to pathogen invasion [43] influencing diarrheal disease in particular [44]. It is unclear what role HIV status had on outbreak dynamics in this study given the barriers in obtaining accurate status information from interviewed patients. However, the high prevalence of HIV infection among the community suggests that this is an important component requiring further investigation. Across Chobe District, a significant percent of patients reporting diarrheal disease were in the 5+ age class (44% and 35% in the dry and wet season outbreaks, respectively). Our survey results did not identify any significant difference by age in patients reporting persistent or chronic diarrhea during outbreak periods (Figure 3). While global surveillance focus is directed at the under-5 age group [45], this may not be appropriate in areas of high HIV prevalence. Distinguishing between infectious and chronic, noninfectious diarrheal disease in HIV infected individuals may be an important challenge to diarrheal disease surveillance in this group.

Interventions directed at reducing HIV transmission may also influence diarrheal disease dynamics. For example, natural maternal immunity, important in fighting water-associated infection, is absent in a great percentage of children in Africa, particularly in Botswana, as HIV-positive mothers are encouraged to use formula rather than breastfeed to reduce mother-to-child transmission of the virus [46]. In Chobe District in 2007, only 52% of children were reported as having been breast-fed [33]. A similar level was reported among participants in this study with only 44% and 63% reported to have been breast-fed in the dry and wet season outbreaks, respectively (Figure 3A). In the large 2006 diarrheal outbreak in Botswana, affected children were less likely to have been breast fed [47]. There is an urgent need to refine our understanding of these interactions and implications to both HIV and diarrheal disease management and public health strategy.

### Malnutrition

The Botswana Government, since the early 1980s, has monitored and provided free supplementary foods to any child with malnutrition [48] or those nutritionally vulnerable, such as orphans associated with the HIV/AIDS [49]. Malnutrition is identified as a critically important prognostic indicator of mortality in diarrheal disease [45]. While malnutrition was not noted in the wet season outbreak, 14% of the under -5 patients were identified in the dry season as suffering from malnutrition by nursing staff filling in survey forms. Nutritional status among children can influence disease susceptibility and diarrheal disease [50]. Consequently, an important focus is directed at ensuring adequate nutrition in early and recovery stages of diarrheal disease [51]. While anorexia can occur as a consequence of diarrheal disease [52], in many places including Botswana, caregivers may also withhold food and milk products, including breast milk, in response to the onset of diarrheal disease in a child [38]. During the dry season outbreak assessed in this study, several mothers with children in the pediatric ward identified withholding food and milk products, although public health educational campaigns actively discourage this practice in diarrheal disease management. Understanding these and other health care behaviors may be critical to public strategy effectiveness and underscore the need to identify locally held beliefs, risk perceptions and behavior, and home-based, health care practices.

### Water shortages and quality

Human health is directly related to the quality and quantity of readily available water, which, in turn, influences exposure to waterborne pathogens, hygiene and sanitation practices, and the occurrence of diarrheal illness [53]. This is particularly true for much of the African continent, where poverty, water quality, and sanitation deficiencies have strongly influenced declining human health levels and, in particular, diarrheal illness [54]. Water shortages appear to be an important common element in both outbreaks across seasons. In Botswana, in general, water shortages occur commonly. This is particularly so in more remote regions as a result of equipment breakdowns, fuel shortages, and HIV/AIDS absenteeism from work related to water services [55]. Many communities suffer chronic disruptions in water delivery that can result in the use of poor water quality sources such as river water, ephemeral pans, and open wells [22]. In a municipal system, water shortages or cessation in delivery may also lead to declines in water quality. In this study, many respondents identified dirt or discoloration in municipal water. In Chobe District, as elsewhere, large centralized water tanks provide water for sections of the municipal system. During shortages, these tanks are quickly drained through local consumption, but retain small quantities of standing water and sediment from previous fillings. Re-suspension of both bacteria and sediment on resumption of delivery may be possible. Shortages, which occur year round, might be more important when cyclic changes occur in water quality related to seasonal hydrological phenomena (rainfall, flooding) identifying potential linkages where humans health and environmental conditions are coupled.

In Chobe District, like many African rural areas, alternate sources of clean water are unavailable during water shortages or periods of poor quality, particularly for the poor where purchasing of bottled water is economically infeasible. Use of unsafe water resources can lead to exposure to waterborne pathogens and diarrheal disease. In our study site, the Chobe River is accessible to most community members living in the District with the exception of Lesoma and Pandamatenga villages (Figure 1). Direct exposure to surface water through swimming, cleaning vegetables, and/or washing laundry was infrequently reported (6.4% and 0% by outbreak period, respectively) among patients and no one reported using the Chobe River directly as a drinking water source. This is an important finding, as the potential use of the Chobe River for drinking and other household uses during water shortages may have been considered an important hypothesized contributor to diarrheal outbreaks.

Recurrent water shortages lead to an increase in water storage practices [56], elevating the potential for post source contamination to occur, an increasing concern in Africa and elsewhere [57]. Water is also stored in containers in order to increase water access where water is not piped into the home (Figure 3A). Storage of water was reported among most patients surveyed. Contamination of stored household water can occur at the source or post collection during utilization by the family [58]. However, while there is great concern regarding the potential for post-collection contamination of water, other comparative studies suggest that source water quality is still more significant than water storage practices in determining water quality and diarrheal disease incidence [58].

### Sanitation

The majority of respondents reported a lack of waterborne sanitation with most reporting the use of pit latrines (Figure 3A) reflective of the community in general (unpublished data). Only one individual reported lack of access and the use of a bush latrine (open air defecation) although lack of sanitation is not uncommon in the study area (unpublished data). It may be that a higher number of individuals did not have access to sanitation facilities but were reluctant to disclose this information during an interview. Previous studies have identified the benefits of sanitation on diarrhea and other diseases with more significant reductions achieved with flush toilets than pit latrines [59]. However, access to flush toilets may not be possible even with heavy government investment in sanitation infrastructure as poorer people are often still unable to identify funds necessary for connection fees and development of associated infrastructure. Simple pit latrines, commonly used in the area may also, however, provide increased excreta access for flies, increasing fly population density and potential fly borne transmission of disease causing diarrheal organisms (reviewed [7]). Heavy rainfall events and overflowing of pit latrines has also been linked to the occurrence of major diarrheal outbreaks where high levels of morbidity and mortality occurred [32].

Pit latrines have also been associated with declines in water quality through bacterial contamination and nitrate leaching of water resources (reviewed in [60]). Saturated soils can facilitate this process with microorganism movement occurring up to several hundred meters through subsurface water flow [61-63]. The complicated manner in which sanitation may influence health in the region is a critical area of future research.

### Appraisal of approach, limitations, and lessons learned

Our study data were available prior to collation of summary patient attendance data and provided insight into the relative magnitude of the outbreak and unique data on the characteristics of patients involved in the respective outbreaks.

The most important elements influencing the effectiveness of this method appeared to be motivation of individual and particularly, senior staff, appropriate variable selection crafted to the area and questions at hand, and the need to keep the instrument short in order to increase its use during outbreak periods where higher patient - staff ratios are experienced. Improved delivery and return of questionnaires and medical staff participation may be enhanced if implemented through existing institutional and hierarchical structures and not externally driven with essentially volunteer contributions, as was the case here. Factors influencing participation were not systematically assessed, however, but such an evaluation will be important to improving the approach and replicating it successfully in other medical facilities. Finally, feedback of study results by the lead author to the involved facility medical staff (doctors, nurses, and laboratory staff) and District health officials contributed to a greater understanding of diarrheal disease dynamics and more positive views of the exercise and resultant investment of time. These latter activities are important components of the study approach, increasing the use of study results and identifying local value in such an exercise.

The methods and data acquired here cannot be used to make any characterizations or inferences regarding the larger Chobe population experiencing diarrheal disease and does not take the place of a case–control study. It does, however, provide a mechanism to evaluate patient characteristics during outbreaks, inform future study hypotheses and approaches, and potentially identify immediate public health interventions. This information is acquired without relying on any additional funding or staffing resources. These latter considerations dominate resource poor settings and contribute to a continued lack of information regarding characteristics of disease outbreaks in these areas.

## Conclusions

This study identifies diarrhea in under-5 in Chobe District, Botswana as a persistent problem with distinct seasonality in diarrheal case incidence. Lack of variation in outbreak variables suggests the possibility of environmental drivers influencing outbreak dynamics and the potential importance of human-environmental linkages in this region. Until more information is available, public health strategy should be directed at securing improved water service to the region and correcting existing water quality deficiencies. Public health education should include increased emphasis on the importance of improved sanitation practices when providing care to household members with diarrhea. While global diarrheal disease surveillance is directed at the under-5 age group, this may not be appropriate in areas of high HIV prevalence where the occurrence of a large immune-compromised population may warrant increased surveillance across age groups.

More emphasis is needed on finding ways to improve our understanding of diarrheal disease in these resource poor settings that acknowledge and engage the persistent human, infrastructural, and economic limitations often identified in these areas. The approach employed in this study provided the first detailed characterization of patients presenting with diarrheal disease during outbreaks and identified an important starting point for immediate intervention and development of working hypotheses for future disease investigations. While data derived from this approach are necessarily limited, they identify critical information on outbreak characteristics in a resource poor setting where data gaps continue and disease incidence is high.

## Competing interests

The authors declare that they have no competing interests.

## Authors' contributions

KA designed the study, collected, and analyzed the data. KA performed the cluster and CART analysis and KA and JKB produced the descriptive statistics. JKB created the study map. KA drafted the manuscript and prepared figures and tables. KA and JKB edited the manuscript content. KA revised the manuscript incorporating reviewer’s comments. All authors read and approved the final manuscript.

## Pre-publication history

The pre-publication history for this paper can be accessed here:

http://www.biomedcentral.com/1471-2458/13/775/prepub
